# Molecular Mechanisms and Epidemiology of Fosfomycin Resistance in *Staphylococcus aureus* Isolated From Patients at a Teaching Hospital in China

**DOI:** 10.3389/fmicb.2020.01290

**Published:** 2020-06-26

**Authors:** Wenya Xu, Tao Chen, Huihui Wang, Weiliang Zeng, Qing Wu, Kaihang Yu, Ye Xu, Xiucai Zhang, Tieli Zhou

**Affiliations:** ^1^Department of Clinical Laboratory, The First Affiliated Hospital of Wenzhou Medical University, Wenzhou, China; ^2^School of Laboratory Medicine and Life Science, Wenzhou Medical University, Wenzhou, China

**Keywords:** fosfomycin, *Staphylococcus aureus*, resistance mechanism, mutation, *tet38*

## Abstract

*Staphylococcus aureus* is a major cause of hospital- and community-acquired infections placing a significant burden on the healthcare system. With the widespread of multidrug-resistant bacteria and the lack of effective antibacterial drugs, fosfomycin has gradually attracted attention as an “old drug.” Thus, investigating the resistance mechanisms and epidemiology of fosfomycin-resistant *S. aureus* is an urgent requirement. In order to investigate the mechanisms of resistance, 11 fosfomycin-resistant *S. aureus* isolates were analyzed by PCR and sequencing. The genes, including *fosA*, *fosB*, *fosC*, *fosD*, *fosX*, and *tet38*, as well as mutations in *murA*, *glpT*, and *uhpT* were identified. Quantitative real-time PCR (qRT-PCR) was conducted to evaluate the expression of the target enzyme gene *murA* and the efflux pump gene *tet38* under the selection pressure of fosfomycin. Furthermore, multilocus sequence typing (MLST) identified a novel sequence type (ST 5708) of *S. aureus* strains. However, none of the resistant strains carried *fosA*, *fosB*, *fosC*, *fosD*, and *fosX* genes in the current study, and 12 distinct mutations were detected in the *uhpT* (3), *glpT* (4), and *murA* (5) genes. qRT-PCR revealed an elevated expression of the *tet38* gene when exposed to increasing concentration of fosfomycin among 8 fosfomycin-resistant *S. aureus* strains and reference strain ATCC 29213. MLST analysis categorized the 11 strains into 9 STs. Thus, the mutations in the *uhpT*, *glpT*, and *murA* genes might be the primary mechanisms underlying fosfomycin resistance, and the overexpression of efflux pump gene *tet38* may play a major role in the fosfomycin resistance in these isolates.

## Introduction

*Staphylococcus aureus* is a kind of facultative anaerobe pathogenic Gram-positive coccus with strong resistance and tolerance to harsh environments (Wang et al., 2020). At present, *S. aureus* has become a significant pathogen of nosocomial infections, such as deep-seated skin and soft tissue infections (SSTI), endocarditis, and other life-threatening severe infections (Mehraj et al., 2016). In recent years, with the widespread use of antibiotics, the emergence of multidrug-resistant (MDR) *S. aureus* has become a major concern (Gatadi et al., 2019). In addition, the lack of effective clinical treatments against MDR *S. aureus* has rekindled the interest of clinicians in fosfomycin. It is an antimicrobial agent that was discovered in *Streptomyces* sp. It exhibits broad-spectrum activity against both Gram-positive and Gram-negative bacteria by inhibiting the peptidoglycan synthesis pathway, which is essential for the synthesis of the cell walls (Shorr et al., 2017). However, the number of fosfomycin-resistant *S. aureus* strains is increasing rapidly (Etienne et al., 1991).

Several mechanisms of fosfomycin resistance have been proposed, including the plasmid-encoded fosfomycin-modifying enzymes (FosA, FosB, FosC, FosD, and FosX) and the acquisition of chromosomal mutations (Nakaminami et al., 2008; Liu et al., 2017; Silver, 2017). Mutations in the MurA target enzyme and transporters (GlpT and UhpT) have been shown to be responsible for fosfomycin resistance (Michalopoulos et al., 2011). Additionally, the overexpression of target enzymes, MurA and Tet38 efflux pump, also contributes to fosfomycin resistance in *S. aureus* (Truong-Bolduc et al., 2018). Notably, there are no reports yet suggesting that fosfomycin can stimulate the expression of the efflux pump gene and mediate drug resistance. However, a few studies on *S. aureus* have described the drug sensitivity and resistance mechanism of fosfomycin in *S. aureus*, although they are not fully understood.

In the present study, we focus on the mutations of the target enzyme MurA, which catalyzes the initial step in the biosynthesis of peptidoglycan and transporters (GlpT and UhpT), as well as the overexpression of *murA* and *tet38* efflux pump in 11 fosfomycin-resistant *S. aureus.* In addition, a strong correlation was established between fosfomycin resistance and efflux pump gene *tet38* overexpression that has not been reported previously. The results of quantitative real-time PCR (qRT-PCR) indicated that the Tet38 efflux pump plays a vital role in fosfomycin resistance by pumping out the drug.

## Materials and Methods

### Bacterial Strains

In 2018, a total of 200 *S. aureus* isolates were obtained from the First Affiliated Hospital of Wenzhou Medical University, a comprehensive teaching hospital in China. The bacteria were identified by matrix-assisted laser desorption/ionization time of flight mass spectrometry (MALDI-TOF MS; BioMérieux, Lyons, France). *S. aureus* ATCC 29213 (American Type Tissue Culture Collection, Manassas, VA, United States) was used as an endogenous control strain in antimicrobial susceptibility testing experiments. The study and consent procedure were approved by the Ethics Committee of the hospital.

### Antimicrobial Susceptibility Testing

The minimum inhibitory concentration (MIC) of fosfomycin for each clinical strain was determined using the agar dilution method, wherein the media were supplemented with glucose-6-phosphate (25 mg/L), according to the recommendations of the Clinical and Laboratory Standards Institute [CLSI], 2018 (Ushanov et al., 2020). The data were interpreted according to the European Committee on Antimicrobial Susceptibility Testing criteria (available at http://www.eucast.org/clinical_breakpoints/) (susceptible, ≤32 mg/L; resistant, ≥64 mg/L), and the fosfomycin-resistant isolates were selected for further investigation. In addition, the MICs of fosfomycin-resistant *S. aureus* to other classes of antibiotics, including oxacillin, erythromycin, ciprofloxacin, levofloxacin, gentamicin, rifampicin, linezolid, vancomycin, and teicoplanin, were detected using the broth microdilution method.

### Detection of Fosfomycin-Resistant Genes

The DNA of fosfomycin-resistant and fosfomycin-susceptible *S. aureus* isolates was extracted using a Biospin Bacterial Genomic DNA Extraction Kit (Bioflux, Tokyo, Japan) and was utilized as the template for PCR amplification of the *fosA*, *fosB*, *fosC*, *fosD*, *fosX*, *glpT*, *uhpT*, *murA*, and *tet38* genes; the primers are listed in Table 1. The PCR products were sequenced by Beijing Genomics Institute Technology Co., Ltd. (Shanghai, China), and the sequences were aligned by BLAST on the NCBI platform^1^. The PCR products of *uhpT*, *glpT*, and *murA* were sequenced to scan for mutations.

**TABLE 1:** Primers used in this study.

Gene	Primer sequences (5′→3′)	Product size (bp)	References
**PCR primers**			
*fosA*	F:GCTGCACGCCCGCTGGAATA	217	Chen et al., 2014
	R:CGACGCCCCCTCGCTTTTGT		
*fosB*	F:CAGAGATATTTTAGGGGCTGACA	312	Chen et al., 2014
	R:CTCAATCTATCTTCTAAACTTCCTG		
*fosC*	F:GGGTTACATGCCCTTGCTCA	354	Chen et al., 2014
	R:AACCCGCACAACGACAGATG		
*fosD*	F: AACTCTAACTTGTGTCCGTCAG	220	Liu et al., 2017
	R: GTGGCTTATGGGTTGCGTTA		
*fosX*	F: ATGATCAGTCATATGACATTTATCG	243	Zhang et al., 2020
	R: ATTTAGCCCCTTGTCGATAACG		
*murA*	F:GCCCTTGAAAGAATGGTTCGT	1600*	NC_002745.2**
	R:GTTACAATACTCGACGCAGGT		
*glpT*	F:TGAATAAAACAGCAGGGCAA	1699*	NC_002745.2**
	R:CACAGCTAGTATGTATAACGAC		
*uhpT*	F:TGTGTTTATGTTCAGTATTTTGGA	1571*	NC_002745.2**
	R:TCTTTCATCTCTTCACGCAC		
*tet38*	F:GCGGATACAACAGCGAGTGA	1353	Truong-Bolduc et al., 2005
	R:TCGACGCACCTAATGGGAAT		
**qRT-PCR primers**			
*gmk*	F:ACTAGGGATGCGTTTGAAGC	122	Chen and Hooper, 2018
	R:TCATGACCTTCGTCCATTGT		
*tet38*	F:TGACAGGTGTGGCTATTGGT	112	Chen and Hooper, 2018
	R:TTGCCTGGGAAATTTAATGC		
*murA*	F:TGTGCACCTTGCAATTGACT	102	G et al., 2019
	R:CCGTTTTATGCATGTTGCAG		

*PCR product including surrounding sequences adjacent to the target gene. **GenBank-EMBL-DDBL accession number.

### Fosfomycin Treatment and Total RNA Isolation

Actively growing *S. aureus* specimens were treated with increasing concentrations of fosfomycin (1/8 MIC, 1/4 MIC, and 1/2 MIC) for 2 h, after which the cells were harvested, and total RNA was extracted using a Bacterial RNA Miniprep Kit (Biomiga, Shanghai, China) according to the manufacturer’s instructions. Then, 1000 ng RNA was used as the template for reverse transcription using a RevertAid First Strand cDNA Synthesis Kit (Thermo Scientific, Waltham, MA, United States) to obtain cDNA.

### Quantitative Real-Time PCR (qRT-PCR)

qPCR was performed on a CFX-96 Touch^TM^ Real-Time PCR system (Bio-Rad, Hercules, CA, United States) using TB Green Premix Ex Taq II (Tli RNase H Plus) (2×) (Takara, Japan), specific primers (Table 1), and 100 ng cDNA as the template. Cycling conditions were as follows: 95°C for 30 s, followed by 40 cycles of 95°C for 5 s and 60°C for 20 s. A melting curve was performed after each run (raising the temperature by 0.5°C/s, from 65 to 95°C). Each sample was run in triplicate, and the means of the Ct values were used for analysis. The relative expression levels of *tet38* and *murA* genes were normalized to the *gmk* reference gene (Chen and Hooper, 2018). The quantification of the target genes was analyzed using the comparative threshold cycle 2^–ΔΔ*Ct*^ method. All experiments were repeated in triplicate independently. The relative expression of the mRNA of the target gene was normalized to that of *S. aureus* ATCC 29213.

### Multilocus Sequence Typing (MLST)

Isolates were screened using a previously described method to detect the following seven housekeeping genes: carbamate kinase (*arcC*), shikimate dehydrogenase (*aroE*), glycerol kinase (*glp*), guanylate kinase (*gmk*), phosphate acetyltransferase (*pta*), triosephosphate isomerase (*tpi*), and acetyl coenzyme A acetyltransferase (*yqiL*) (Enright and Spratt, 1999). The sequences of the PCR products were compared with those available from the MLST website^2^ for *S. aureus*. Also, the allelic number was determined for each sequence.

### Planktonic Growth Assay

The planktonic growth rates of 8 *tet38*-overexpressed *S. aureus* isolates were determined as described previously (Wijesinghe et al., 2019), with some modifications. Briefly, 8 *tet38*-overexpressed isolates and ATCC 29213 standard cell suspensions were prepared by adjusting the turbidity of suspension to 0.5 McFarland standard in sterile saline. Then, 200 μL of each suspension was inoculated in 20 mL sterile LB broth containing fosfomycin in 0, 1/8 MIC, 1/4 MIC, and 1/2 MIC, respectively, for growth at 37°C and 180 rpm for 24 h. The growth rate of the planktonic bacteria was determined by measuring the optical density (OD) of the suspension in each well of the 96-well plate at 600 nm at 2-h intervals for 24 h using a microtiter plate reader (BioTek, United States). The growth curve was generated in triplicate for each experiment. ATCC 29213 served as the control strain.

### Statistical Analysis

The relative expression of *murA* and *tet38* was compared using Student’s *t*-test, and *P-*value < 0.05 was considered to be statistically significant.

## Results

### Susceptibility to Fosfomycin and Other Types of Antibiotics

The susceptibility to fosfomycin of 200 *S. aureus* isolates was determined by the agar dilution method using glucose-6-phosphate (25 mg/L). The results showed that 5.5% (11/200) of the isolates were resistant to fosfomycin. Also, resistance to other antibiotics was determined (Table 2); 81.8% (9/11) of the isolates displayed resistance to erythromycin, while 72.7% (8/11) belonged to MDR *S. aureus*.

**TABLE 2:** Characteristics and resistance spectrum of fosfomycin-resistant *S. aureus* strains.

Isolates	ST type	FOM	OXA	ERY	CIP	LVX	GEN	RIF	LNZ	VAN	TEC
JP3187	5	256^*R*^	>128^R^	32^R^	>256^R^	32^R^	4	<0.03	2	2	4
JP3189	4539	64^R^	>128^R^	64^R^	64^R^	32^R^	16^R^	>16^R^	1	1	4
JP3212	5	256^R^	>128^R^	64^R^	128^R^	32^R^	4	<0.03	2	2	8
JP3235	5708	64^R^	>128^R^	1	128^R^	32^R^	64^R^	>16^R^	2	1	4
JP3244	7	128^R^	0.5	64^R^	0.25	0.25	<0.125	<0.03	2	1	0.5
JP3505	4739	512^R^	0.5	1	2	1	<0.125	<0.03	4	2	0.5
JP3535	5	64^R^	>128^R^	16^R^	128^R^	16^R^	0.25	<0.03	4	1	2
JP3539	59	64^R^	8^R^	64^R^	0.5	0.25	0.25	<0.03	4	2	1
JP3589	1	64^R^	0.25	64^R^	2	1	2	<0.03	2	2	1
JP3592	239	256^R^	>128^R^	64^R^	128^R^	64^R^	<0.125	>8^R^	4	1	1
JP3600	965	64^R^	0.5	64^R^	4^R^	1	0.5	<0.03	2	2	2

*FOM, fosfomycin; OXA, oxacillin; ERY, erythromycin; CIP, ciprofloxacin; LVX, levofloxacin; GEN, gentamicin; RIF, rifampicin; LNZ, linezolid; VAN, vancomycin; TEC, teicoplanin. Superscript “R” indicates resistance.*

### Molecular Mechanisms of Fosfomycin-Resistant Isolates

**FIGURE 1:**
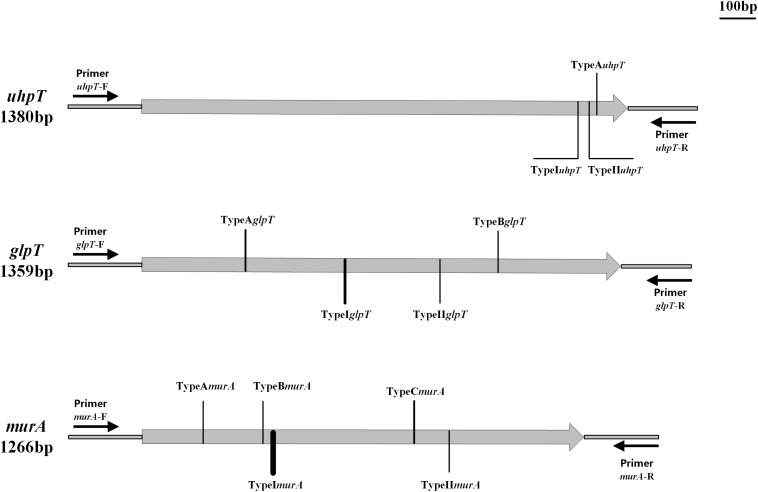
Types and positions of mutations in *uhpT*, *glpT*, and *murA* genes. TypeA*_*uhpT*_*: T1369 G; TypeI*_*uhpT*_*: G1364A; TypeII*_*uhpT*_*: T1368G. TypeA_*glp*_*_*T*_*: C299T; TypeB_*glp*_*_*T*_*: G1064A; TypeI_*glp*_*_*T*_*: G583A; TypeII_*glp*_*_*T*_*: T829G. TypeA*_*murA*_*: G187A; TypeB*_*murA*_*: G349T; TypeC*_*murA*_*: G770A; TypeI*_*murA*_*: C371G; and TypeII*_*murA*_*: A873T.

Strains carrying the *fosA*, *fosB*, *fosC*, *fosD*, or *fosX* gene were not found in the current study (Table 3). Based on the classification method of Fu et al. (2015) we named the sense mutations as TypeA, TypeB, and TypeC according to the amino acid sequence, and the nonsense mutations were named as TypeI, TypeII, and TypeIII; the subscripts represent different genes (Fu et al., 2015). Three distinct mutations were detected in the *uhpT* gene of the 11 fosfomycin-resistant *S. aureus* isolates and the corresponding sensitive strains. Mutation TypeA*_*uhpT*_*, found in JP3212, resulted in an amino acid substitution at position 457 (Leu457Val) of UhpT. Conversely, the other two mutations (TypeI–II*_*uhpT*_*), which resulted in distinct amino acid substitutions within the UhpT protein, were identified in fosfomycin-sensitive isolates, although one mutation (TypeII*_*uhpT*_*) was also found in fosfomycin-resistant *S. aureus* (Figure 1 and Table 3).

**TABLE 3:** Characteristics and amino acid substitutions in fosfomycin-resistant and fosfomycin-sensitive *S. aureus.*

Strains	Type	*tet38*	*fos*					Amino acid substitution		
				
			*fosA*	*fosB*	*fosC*	*fosD*	*fosX*	*uhpT*	*glpT*	*murA*
JP3187	R	+	–	–	–	–	–	None	TypeA*_*glpT*_*	TypeI*_*murA*_*
JP3189	R	+	–	–	–	–	–	None	None	TypeC*_*murA*_*
JP3212	R	+	–	–	–	–	–	TypeA*_*uhpT*_*	None	TypeI*_*murA*_*
JP3235	R	+	–	–	–	–	–	None	None	TypeI*_*murA*_* TypeC*_*murA*_*
JP3244	R	+	–	–	–	–	–	None	None	TypeI*_*murA*_* TypeC*_*murA*_*
JP3505	R	+	–	–	–	–	–	None	TypeI*_*glpT*_*	TypeA*_*murA*_* TypeI*_*murA*_*
JP3535	R	+	–	–	–	–	–	None	TypeI*_*glpT*_* TypeB*_*glpT*_*	TypeI*_*murA*_*
JP3539	R	+	–	–	–	–	–	None	TypeA*_*glpT*_* TypeI*_*glpT*_*	TypeI*_*murA*_* TypeII*_*murA*_*
JP3589	R	+	–	–	–	–	–	None	None	TypeI*_*murA*_* TypeB*_*murA*_*
JP3592	R	+	–	–	–	–	–	None	None	TypeI*_*murA*_* TypeC*_*murA*_*
JP3600	R	+	–	–	–	–	–	TypeII*_*uhpT*_*	TypeI*_*glpT*_*	TypeI*_*murA*_*
JP3200	S	+	–	–	–	–	–	None	None	None
JP3203	S	+	–	–	–	–	–	TypeI*_*uhpT*_*	None	None
JP3277	S		–	–	–	–	–	None	None	None
JP3230	S	+	–	–	–	–	–	None	None	None
JP3240	S	+	–	–	–	–	–	TypeII*_*uhpT*_*	TypeI*_*glpT*_* TypeII*_*glpT*_*	None
JP3245	S	+	–	–	–	–	–	None	None	None
JP3502	S	+	–	–	–	–	–	None	None	TypeI*_*murA*_* TypeII*_*murA*_*
JP3512	S	+	–	–	–	–	–	None	None	None
JP3518	S	+	–	–	–	–	–	None	None	TypeI*_*murA*_*
JP3520	S	+	–	–	–	–	–	None	TypeI*_*glpT*_*	TypeI*_*murA*_*
JP3522	S	+	–	–	–	–	–	None	None	TypeI*_*murA*_*

*+, carries the corresponding gene; –, does not carry the corresponding gene; None: nonsense mutation; TypeA*_*uhpT*_*: T1369 G; TypeI*_*uhpT*_*: G1364A; TypeII*_*uhpT*_*: T1368G; TypeA_*glp*_*_*T*_*: C299T; TypeB_*glp*_*_*T*_*: G1064A; TypeI_*glp*_*_*T*_*: G583A; TypeII_*glp*_*_*T*_*: T829G; TypeA*_*murA*_*: G187A; TypeB*_*murA*_*: G349T; TypeC*_*murA*_*: G770A; TypeI*_*murA*_*: C371G; TypeII*_*murA*_*: A873T.*

Moreover, four different mutations were detected in the *glpT* gene (TypeA–B*_*glpT*_* and TypeI–II*_*glpT*_*). Notably, TypeB*_*glpT*_*, found in the fosfomycin-resistant isolates JP3535, produced a premature stop codon within the *glpT* coding sequence at position 355 (Trp335Ter), thereby resulting in truncated proteins. In addition, TypeII*_*glpT*_* was detected only in the fosfomycin-sensitive isolates (Figure 1 and Table 3).

Of the 11 fosfomycin-resistant isolates, 6 contained one of the three different mutations (TypeA–C*_*murA*_*) in the *murA* gene. TypeA–C*_*murA*_*, which resulted in distinct amino acid substitutions within the MurA protein at positions 63 (Ala63Thr), 117 (Gly117Trp), and 257 (Gly257Asp), could only be found in fosfomycin-resistant isolates, and two mutations (TypeI–II*_*murA*_*) could be found in both fosfomycin-resistant and fosfomycin-susceptible *S. aureus* (Figure 1 and Table 3). Moreover, only one sense mutation was present in each fosfomycin-resistant *S. aureus* isolate.

### Expression Analysis of *murA* and *tet38*

qRT-PCR revealed significant differences in the expression of *murA* between resistant and susceptible groups of *S. aureus* as compared with *S. aureus* ATCC 29213 (*P* < 0.05) (Table 4). The data showed that the average expression level of *murA* gene decreased by 0.7-fold in fosfomycin-resistant and fosfomycin-susceptible *S. aureus* isolates. In addition, compared with the fosfomycin-susceptible *S. aureus*, the expression of *murA* in the resistance isolates was not significantly higher.

**TABLE 4:** Relative expression of target enzyme gene *murA* and efflux pump gene *tet38* in fosfomycin-susceptible and fosfomycin-resistant *S. aureus*.

Strains	Relative expression level of *murA*^a^ (mean ± SD)	Relative expression level of *tet38*^a^ (mean ± SD)
S1	0.77 ± 0.15	0.77 ± 0.13
S2	1.45 ± 0.10	1.48 ± 1.02
S3	0.40 ± 0.05	0.71 ± 0.21
S4	0.48 ± 0.01	1.55 ± 0.33
S5	0.48 ± 0.06	0.98 ± 0.35
JP3187	0.75 ± 0.09	1.71 ± 0.93
JP3189	0.35 ± 0.01	0.71 ± 0.25
JP3212	0.47 ± 0.01	**21.6 ± 5.75**
JP3235	1.47 ± 0.14	0.92 ± 0.34
JP3244	0.21 ± 0.04	1.24 ± 0.15
JP3505	0.23 ± 0.04	**2.65 ± 1.04**
JP3535	0.34 ± 0.03	**2.74 ± 0.37**
JP3539	0.39 ± 0.05	1.71 ± 0.48
JP3589	0.42 ± 0.08	0.54 ± 0.23
JP3592	0.40 ± 0.07	**143.36 ± 2.05**
JP3600	1.52 ± 0.23	**24.59 ± 0.17**

*S1–S5 represent the 5 fosfomycin-susceptible *S. aureus* strains. ATCC 29213 served as the control strain. ^*a*^The relative gene expression with more than 2-fold change compared with ATCC 29213 after fosfomycin induction is shown in bold.*

However, the results (Table 4) indicated that compared with that in ATCC29213 and susceptible isolates, the expression level of efflux pump gene *tet38* in JP3212, JP3535, JP359, and JP3600 was elevated. Notably, the level of the *tet38* gene in JP3212, JP3535, JP3592, and JP3600 was altered markedly (21. 60-, 2. 74-, 143. 36-, and 24.59-fold) as compared with that in ATCC29213 (Table 4).

### Exposure to Fosfomycin Resulted in Increased Expression of *tet38* Efflux Pump Genes Among Some Resistant Isolates

**FIGURE 2:**
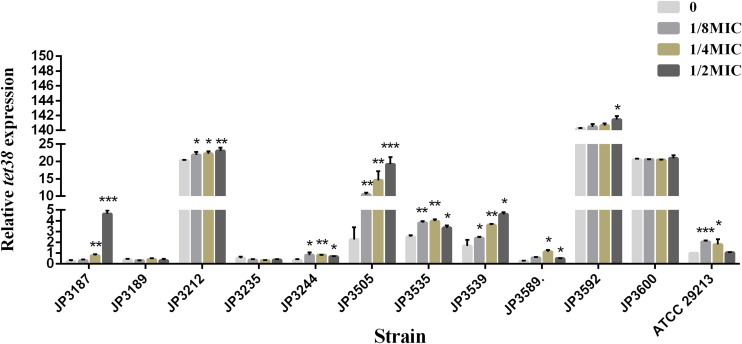
The relative expression of efflux pump gene *tet38* in fosfomycin-resistant *S. aureus* exposed to 0, 1/8 MIC, 1/4 MIC, and 1/2 MIC concentrations. Bars indicate the mean values, and asterisks denote the significant difference of expression (*P* < 0.05).

The expression of *tet38* in the presence of increasing amounts of fosfomycin with 0, 1/8 MIC, 1/4 MIC, and 1/2 MIC concentrations was determined by qRT-PCR. Notably, the expression of the *tet38* gene in JP3187, JP3212, JP3244, JP3505 JP3535, JP3539, P3589, JP3592, and ATCC 29213 was upregulated with the increase in fosfomycin concentration as compared with the 0 MIC strains (Figure 2 and Table 4). Also, 4.63-fold and 6.42-fold increases were noted in the expression of *tet38* in JP3505 cells treated with 1/8 MIC (64 mg/L) and 1/4 MIC (128 mg/L) fosfomycin, respectively, as compared with that in cells that were not treated with fosfomycin (Table 5). A further 8.46-fold increase was observed in those treated with 1/2 MIC (256 mg/L) fosfomycin.

**TABLE 5:** Relative expression of efflux pump gene *tet38* in fosfomycin-resistant *S. aureus* exposed to different concentrations of fosfomycin.

Strains	The relative expression level of *tet38*^a^ (mean ± SD)			
	
	0 MIC	1/8 MIC	1/4 MIC	1/2 MIC
JP3187	0.250.09	0.320.08	**0.77 ± 0.10**	**4.64 ± 0.25**
JP3189	0.380.08	0.320.01	0.430.07	0.320.13
JP3212	20.320.10	21.890.69	22.220.56	23.020.8
JP3235	0.530.12	0.360.08	0.330.03	0.370.06
JP3244	0.350.06	**0.81 ± 0.20**	**0.83 ± 0.01**	0.680.03
JP3505	2.270.91	**10.51 ± 0.38**	**14.57 ± 2.12**	**19.21 ± 1.65**
JP3535	2.500.12	3.820.12	3.950.14	3.340.17
JP3539	1.670.45	2.400.08	**3.60 ± 0.08**	**4.60 ± 0.14**
JP3589	0.270.02	**0.57 ± 0.06**	**1.13 ± 0.14**	0.510.01
JP3592	140.270.05	140.480.30	140.650.23	141.470.36
JP3600	20.580.20	20.620.04	20.470.08	20.930.72

*^*a*^The relative gene expression with more than 2-fold change compared with 0 MIC after fosfomycin induction is shown in bold.*

### Molecular Typing

The 11 fosfomycin-resistant *S. aureus* specimens were categorized into 9 STs (Table 2): ST1 (*n* = 1), ST5 (*n* = 3), ST59 (*n* = 1), ST7 (*n* = 1), ST239 (*n* = 1), ST965 (*n* = 1), ST4539 (*n* = 1), ST4739 (*n* = 1), and a new ST that was found in the current study (ST 5708) (*n* = 1).

### Growth Rate

**FIGURE 3:**
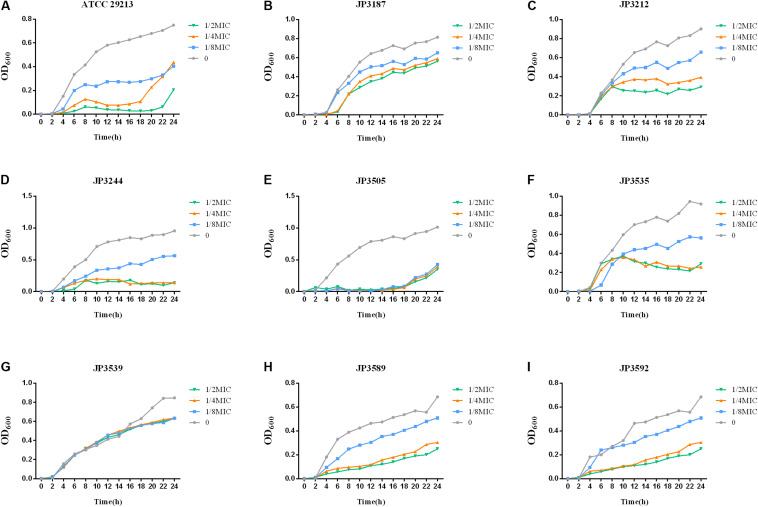
Growth curves at different fosfomycin concentrations in *tet38*-overexpression *S. aureus*. The values shown are the average of three independent experiments. Different colors of lines represent different concentrations of fosfomycin. **(A)** Growth of control strain ATCC 29213. **(B–I)** Growth of 8 *tet38*-overexpression isolates.

In order to gain quantitative insight into the fitness cost imposed by *tet38*-overexpressed isolates, the growth curves of 8 *tet38*-overexpressed *S. aureus* were recorded. We identified a fitness cost after fosfomycin induction. The growth of 8 *tet38*-overexpression strains was inhibited in LB at a subinhibitory concentration of fosfomycin (Figure 3).

## Discussion

Due to the unique mechanisms of action, fosfomycin exhibits significant antimicrobial activity against a broad spectrum of pathogens, including *S. aureus* (Goto, 1977). A review described that the susceptibility of *S. aureus* to fosfomycin ranged from 33.2% to 100% in the nine available studies [odds ratio (OD) = 91.7%, 95% confidence interval (CI): 88.7–94.9%] (Vardakas et al., 2016). In the current study, the susceptibility rate of fosfomycin in *S. aureus* was 94.5% (189/200). However, the prevalence of fosfomycin resistance in clinical isolates of *S. aureus* has been reported with increasing frequency in many areas (Del Rio et al., 2014; Mihailescu et al., 2014; Shi et al., 2014).

The resistance mechanism of fosfomycin in Gram-negative bacteria has been widely reported; also, in a previous study, we reported the resistance of fosfomycin in ESBL-producing *Escherichia coli* (Bi et al., 2017). Fosfomycin enters the cell via two transporters, GlpT and UhpT, and mutations or insertions in *glpT* and/or *uhpT* genes result in the loss of function (Takahata et al., 2010). According to the study by Castaneda-Garcia et al. (2009), *glpT* inactivation played an essential role in the resistance to fosfomycin in *Pseudomonas aeruginosa* (Castaneda-Garcia et al., 2009). The *murA* gene is also closely related to fosfomycin resistance (Takahata et al., 2010; Couce et al., 2012). In addition, fosfomycin activity can be inhibited via the catalytic activity of FosA, FosB, and FosC, respectively (Garcia et al., 1995; Lee et al., 2012).

Among Gram-positive bacteria, the resistance mechanism of fosfomycin is rarely reported. In the current study, none of the resistant strains carried the *fosA*, *fosB*, *fosC*, *fosD*, or *fosX* gene, indicating that these genes might not be the primary factors mediating the resistance of *S. aureus* against fosfomycin. Other studies have shown that the prevalence of fosfomycin resistance genes (*fosA*, *fosB*, and *fosC*) was not the predominant factor contributing to resistance in *S. aureus* (Xu et al., 2017). In addition, a total of 12 mutations were found in 11 strains of fosfomycin-resistant *S. aureus* by sequencing analysis. Of these, 3 were detected in *uhpT*, and TypeA*_*uhpT*_* was carried only by fosfomycin-resistant strain JP3212, while TypeI*_*uhpT*_* and TypeII*_*uhpT*_* were found in both fosfomycin-resistant and fosfomycin-sensitive strains, which likely did not contribute to fosfomycin resistance. Within the *glpT* gene of 11 drug-resistant strains, 2 mutations TypeA–B*_*glpT*_* were observed only in the drug-resistant strains. On the other hand, mutation TypeI*_*glpT*_* was widely detected in both drug-resistant and susceptible strains. Intriguingly, TypeB*_*glpT*_*, which generated a stop codon (TA_1064_G) at position 335 (Figure 1), was harbored in JP3535. Also, we found a mutation (TypeII*_*glpT*_*) merely in the fosfomycin-sensitive strains. Of the five *murA* gene mutations found in the drug-resistant strains, TypeI*_*murA*_* and TypeII*_*murA*_* could also be found in susceptible strains, and the remaining three mutations (TypeA–C*_*murA*_*) were found only in drug-resistant strains (TypeA*_*murA*_*: JP3505; TypeB*_*murA*_*: JP3589; TypeC*_*murA*_*: JP3189, JP3235, JP3244; JP3592). In addition, TypeI*_*murA*_* was also found in fosfomycin-resistant strains (10/11). Among the above mutations, four mutation sites, TypeA*_*glpT*_*, TypeB*_*glpT*_*, TypeC*_*murA*_*, and TypeII*_*murA*_*, were consistent with those reported (Fu et al., 2015). We also found that the frequency of *murA* mutation in *S. aureus* was high, which might play a major role in mediating fosfomycin resistance, which needs an in-depth investigation.

Although several studies have mentioned that the overexpression of the *murA* gene can greatly increase the MICs of fosfomycin, the difference in *murA* expression between fosfomycin-sensitive and fosfomycin-resistant *S. aureus* has not been reported (Garcia et al., 1995; Olesen et al., 2014). In the current study, the results of qRT-PCR revealed a significant difference between the fosfomycin-resistant and fosfomycin-susceptible *S. aureus* with respect to the expression of *murA* as compared with that of *S. aureus* ATCC 29213. However, statistical differences could not be detected between two types of strains (Table 4), indicating that the overexpression of target gene *murA* has no role in conferring fosfomycin resistance in the strains identified in this study. Interestingly, some resistant strains showed a downward trend in the expression of *murA*, suggesting that the role of the *murA* gene in fosfomycin needs to be studied further.

Recent studies have shown that the *tet38* gene exerts a specific effect on fosfomycin resistance. According to the study by Truong-Bolduc, the overexpression of *tet38* resulted in a fourfold increase in the MIC of fosfomycin compared with that of the parent strain (Truong-Bolduc et al., 2018). The results of the current study showed that the expression of the *tet38* efflux pump gene in fosfomycin-resistant strains JP3212, JP3535, JP3592, and JP3600 was significantly higher than that in the control strain ATCC29213 and the susceptible strains (*P* = 0.007, *P* = 0.002, *P* < 0.001, *P* < 0.001, respectively). Furthermore, under the treatment of 0, 1/8 MIC, 1/4 MIC, and 1/2 MIC with fosfomycin, we found that the expression of efflux pump gene *tet38* was upregulated in most resistant isolates, even in the reference strain ATCC 29213. Although nonsense mutation was detected in *uhpT*, *glpT*, and *murA* genes, the level of *tet38* in JP3600 was high even without the drug, which might explain the resistance to fosfomycin. This phenomenon suggests that the *tet38* efflux pump plays a critical role in mediating fosfomycin resistance. Reportedly, abscess and other factors can promote the expression of *tet38* (Chen and Hooper, 2018), and the current study has shown that the stimulation of the drug also enhances the expression of the efflux pump, albeit the specific mechanism remains to be studied further. Moreover, the overexpression of *tet38* can also lead to changes in the cost of bacterial fitness. Some studies have demonstrated that the global regulator MgrA functions as a direct regulator of *tetR21*, which is a TetR-like regulator of the *tet38* efflux pump gene. TetR21 acts as a repressor of tet38 expression and may also regulate the expression of other bacterial resistance determinants (Truong-Bolduc et al., 2005, 2015). We speculated that the high expression of the *tet38* gene in *S. aureus* might be related to the regulation of TetR-like regulator TetR21 and the global regulator MgrA. We will also continue to focus on these phenomena in other bacteria in future studies.

MLST analyses designated three fosfomycin-resistant *S. aureus* isolated to ST5. Combined with drug sensitivity, we found that the ST5 resistant strains were resistant to at least five antibiotics. Among 11 fosfomycin-resistant strains, 72.7% were MDR strains, and further follow-up treatment was essential. Wu et al. (2018) reported that ST5 and ST239 strains were usually resistant to fosfomycin and constituted the predominant HA-MRSA clones in China. The new sequence type found in the resistant strain has been submitted to the repository (see text footnote 2).

## Conclusion

A total of 11 fosfomycin-resistant strains were screened out from 200 *S. aureus* isolates, and the mechanism was explored. Our findings indicated that *fosA*, *fosB*, *fosC, fosD*, and *fosX* genes might not be the major resistant mechanism of *S. aureus* to fosfomycin. The mutations within the *glpT*, *uhpT*, and *murA* genes might play a critical role in conferring fosfomycin resistance. However, the role of overexpression of *murA* in fosfomycin resistance needs to be discussed further in *S. aureus*. Also, the phenomenon of overexpression in the *tet38* gene under a subinhibitory concentration of fosfomycin needs to be investigated further.

## Data Availability Statement

The datasets generated for this study are available on request to the corresponding author.

## Author Contributions

WX conducted the experiments, analyzed the data, and wrote the manuscript. TC participated in experiments and writing. HW and WZ provided fosfomycin-resistant strains and participated in the analysis of results. KY and QW participated in the analysis of the results. TZ helped to design the study. YX and XZ designed the study and corrected the manuscript. All authors read and approved the manuscript.

## Conflict of Interest

The authors declare that the research was conducted in the absence of any commercial or financial relationships that could be construed as a potential conflict of interest.

## Footnotes

1 http://blast.ncbi.nlm.nih.gov/Blast.cgi

2 http://www.mlst.net
